# Establishment of Highly Efficient Plant Regeneration, Callus Transformation and Analysis of *Botrytis cinerea-*Responsive PR Promoters in *Lilium brownii* var. *viridulum*

**DOI:** 10.3390/plants12101992

**Published:** 2023-05-16

**Authors:** Yongyao Fu, Liling Shu, Hanyi Li, Xingming Zhang, Xuan Liu, Zhengying Ou, Xiaomeng Liang, Xiangying Qi, Liping Yang

**Affiliations:** 1 School of Advanced Agriculture and Bioengineering, Yangtze Normal University, Chongqing 408100, China; 2 School of Life Sciences, Yan’an University, Yan’an 716000, China; 3 College of Biology and Food Engineering, Chongqing Three Gorges University, Chongqing 404020, China

**Keywords:** *Lilium brownii* var. *viridulum*, plant regeneration, callus, genetic transformation, *Botrytis cinerea*

## Abstract

*Lilium brownii* var. *viridulum*, commonly called Longya lily, is a well-known flower and vegetable plant in China that has poor tolerance to *Botrytis* fungal disease. The molecularimprovement has mainly been restricted to an efficient regeneration and transformation system. In this study, the highly efficient regeneration of Longya lily was established through the optimization of embryogenic callus, adventitious shoot and rooting induction. The major factors influencing transformation (antibiotics, *Agrobacterium* concentration, infection time, suspension solution and coculture medium) were examined. The expression responses of PR promoters (*ZmPR4* and *BjCHI1*) to *B. cinerea* were assessed in transgenic calli. The results showed that Murashige and Skoog (MS) medium with 1.0 mg·L^−1^ picloram (PIC) and 0.2 mg·L^−1^ 1-naphthaleneacetic acid (NAA) under light conditions and MS with 0.5 mg·L^−1^ 6-benzylaminopurine (6-BA) and 1.0 mg·L^−1^ NAA under darkness were optimal for embryogenic callus induction (64.67% rate) and proliferation (3.96 coefficient). Callus inoculation into MS containing 2.0 mg·L^−1^ thidiazuron (TDZ), 0.4 mg·L^−1^ NAA, 1.0 mg·L^−1^ TDZ and 0.5 mg·L^−1^ NAA led to shooting induction (92.22 of rate) and proliferation (3.28 of coefficient) promotion, respectively. The rooting rate reached 99.00% on MS with 0.3 mg·L^−1^ NAA. Moreover, a transformation rate of 65.56% was achieved by soaking the callus in *Agrobacterium* at an OD_600_ of 0.4 for 10 min in modified MS without NH_4_NO_3_ as the suspension solution and coculture medium before selecting 75 mg·L^−1^ hygromycin and 300 mg·L^−1^ cefotaxime. Only the *BjCHI1* promoter was obviously expressed in transgenic calli. These results could facilitate the generation of Longya lily transgenic plants with improved *B. cinerea* resistance.

## 1. Introduction

*Lilium brownii* var. *viridulum* Baker, a perennial bulbous herb, is a mutant variety of *L. brownii* F. E. Brown ex Miellez belonging to *Lilium* in the Liliaceae. It is generally called the Longya lily, meaning dragon’s teeth in Chinese, in producing areas because the bulbs are composed of many thick scales, with broad-brimmed bottoms and pointed and curved tops [1]. Longya lily has a very long history of cultivation in China and can be used as a culinary, medicinal, and ornamental plant. To our knowledge, it was originally produced mainly in the counties of Wanzai, Taihe, Yanzhou and Gao’an in Jiangxi Province; in Longhui, Anhua and Longshan in Hunan Province; and in Yongfu, Ziyuan and Liuzhou in Guangxi Province. By the end of 2015, the cultivation area for the Longya lily reached 125 thousand mu, with an output of 80,000 tons. It has become one of the three major edible lilies in China [1,2]. However, the occurrence of bulb degeneration and fungal disease has become increasingly serious due to vegetative reproduction and obstacles associated with continuous cropping in fields. *Botrytis cinerea*, one of the top ten fungal phytopathogens, causes serious economic losses during lily production and postharvest storage [3,4]. When lily plants are infected with *B. cinerea*, the leaves usually shrink, the floral organs become brown and rotten, and even the stems become black. In the end, the yield of lily bulbs is reduced, and their quality is lost. At present, the control of *Botrytis* disease is largely dependent on bulb disinfection before planting and pesticide spraying during cultivation. Nevertheless, the application of chemicals can cause a number of problems, including water pollution, pesticide residues and bacterial resistance. The cultivation and application of new disease-resistant cultivars by transgenic molecular breeding technology has become one of the most economical, effective, and environmentally friendly strategies for the prevention and treatment of this disease [5]. Therefore, the establishment of highly efficient plant regeneration and genetic transformation methods for Longya lily has become an important project.

In contrast to other transgenic methods, *Agrobacterium*-mediated genetic transformation is a common and widely used method in plant transformation with some advantages, such as lower costs, simpler operations and fewer transgene copies [6,7]. In recent decades, some progress has been made in achieving high levels of plant generation and *Agrobacterium*-mediated genetically modified lilies containing resistance to different diseases of lily. For example, shoot induction and plant regeneration have been established, and transgenic *L. longiflorum* plants have been produced by *Agrobacterium*-mediated transformation [8]. *Lilium* cv. Acapulco was transformed with a defective cucumber mosaic virus replicase gene (*CMV2-GDD*) to obtain transgenic lines that were resistant to the CMV virus [9], and the transgenic *Lilium* ‘Star Gazer’ was generated by overexpressing the rice *RCH10* chitinase gene that confers resistance to *B. cinerea* [10]. The reproduction of *L. pumilum* was achieved through organogenesis and somatic embryogenesis [11], and highly efficient transformation systems were successfully established [12]. Most recently, a regeneration and genetic transformation system was established for Oriental lily ‘Siberia’ and ‘Sorbonne’, and the ‘Aladdin‘ *LaKNOX1* gene and *L. lancifolium* cold resistance gene *LlNAC2* were transformed to obtain transgenic lily plants [13]. However, at present, a highly efficient regeneration system and *Agrobacterium*-mediated transformation have not been established for Longya lily.

In almost all transformed plants, the CaMV35S promoter has usually been selected to drive foreign gene expression. Genes driven by this promoter are expressed in all plant tissues during various stages of development, which in some cases causes adverse effects on plant growth [14]. In particular, when disease resistance-related genes are driven by the CaMV35S promoter, plant cells are still in their defense mode, generating an immune response [15], which has negative effects on plant morphology, quality and yield [16,17,18]. Another type of promoter alternative to the CaMV35S promoter has been positive for transgenic improvement in *Lilium*. Pathogenesis-related protein (PR) promoters can be quickly activated by infection with bacterial or fungal pathogens and restrict transgene expression to infection sites. Thus, it has been universally used to generate transgenic plant resistance to pathogens [19,20,21]. For example, the *Zea mays ZmPR4* promoter was used to generate transgenic rice that was resistant to *Magnaporthe grisea* [22]. In addition, this promoter was also responsive to *Rhizoctonia solani* induction when expressed in wheat calli [23]. In transgenic *Arabidopsis thaliana* and tobacco, the *Brassica juncea BjCHI1* promoter was responsive to the induction of jasmonic acid and *B. cinerea* [24,25]. However, it is unclear whether these two promoters could be responsive to *B. cinerea* in Longya lily.

The receptors for plant regeneration and transformation in *Lilium* may be calli [26,27], leaf blades [28], bulblet scales [29], shoot segments [8], nodal stem sections [30], bulblet scale slices [31], etc. Embryogenic calli are thought to be ideal receptors for efficient plant regeneration and the production of transgenic lily plants [12,13,32,33]. Herein, a highly efficient regeneration system and *Agrobacterium*-mediated transformation are established based on inducible embryogenic calli in Longya lilies for the first time. Furthermore, the expression responses of the *ZmPR4* and *BjCHI1* promoters to *B. cinerea* were identified in transgenic calli. These results provide a resource for the improvement of disease-resistant Longya lily by molecular breeding.

## 2. Materials and Methods

### 2.1. The Induction and Proliferation of Embryogenic Calli

The bulbs of Longya lily with a 14–16 cm perimeter were collected from the Chongqing Institute of Medicinal Plant Cultivation in Nanchuan District, Chongqing, China. Bulb scales were used for the primary culture on an MS basic medium containing 2.0 mg·L^−1^ 6-benzylaminopurine (6-BA), 0.2 mg·L^−1^ 1-naphthaleneacetic acid (NAA) and 30 g·L^−1^ sucrose. Sterilization was performed according to the methods described by Fu et al. (2020). The scales from in vitro regeneration bulblets at approximately 45 days were used for embryogenic callus induction on an MS medium supplemented with different combinations of plant growth regulators, including picloram (PIC) (0.5, 1.0 and 2.0 mg·L^−1^) and NAA (0.2, 0.5 mg·L^−1^) in accordance with the methods of previous reports [11]. The explants were cultured at 23 ± 2 °C in the dark or light/dark photoperiod (5000–6000 lx, 14 h/10 h) for 30 days, and the growth state was observed every week. Each experiment was repeated three times. To obtain enough callus, an original callus, approximately 0.8 cm^3^ in size, was subcultured on an MS medium with different concentration combinations of 6-BA (0.1, 0.5 mg·L^−1^) and NAA (0.1, 0.5 and 1.0 mg·L^−1^). A total of 50 calli were cultured in flasks, and each experiment was repeated three times. The callus proliferation number, multiplication index, callus texture and color were evaluated for each medium. All calli were cultured and recorded as described above.

### 2.2. Shoot Induction, Proliferation and Rooting Induction

The same size (0.5 cm^3^) of callus was cultured in an MS medium with different combinations of plant growth regulators, including thidiazuron (TDZ) at 0.5, 1.0, and 2.0 mg·L^−1^ and NAA at 0.2 and 0.4 mg·L^−1^, for shoot induction. The growth status was observed at 0, 15, 30, and 45 days. The number of shooting calli, the total shooting number, and shoot features were evaluated for each medium at 45 days. Similar shoots were cultured in an MS medium with different combinations of plant growth regulators, including TDZ at 0.5, 1.0, and 2.0 mg·L^−1^ and NAA at 0.2 and 0.5 mg·L^−1^, for proliferation. The growth status, proliferated shoot number, total shoot proliferation number and shoot features were recorded for each medium as described above. For root induction, a few shoots were cultured in an MS or 1/2 MS medium supplemented with different combinations of 6-BA (0.2, 0.3, 0.5 mg·L^−1^) and NAA(0.2, 0.3, 0.5 mg·L^−1^). The number of root shoots, the total induced root number and root features were recorded for each medium at 30 and 45 days. The culture conditions were under light conditions as described in Section 2.1 on callus induction.

### 2.3. Callus Sensitivity to Different Antibiotics

Uniform callus from proliferation was cultured on the best proliferation medium supplemented with different concentrations of cefotaxime (0, 100, 200, 300, 400 mg L^−1^), kanamycin (0, 50, 75, 100, 200, 300 mg L^−1^), and hygromycin (0, 25, 50, 75 and 100 mg L^−1^). A total of 30 calli were tested at each concentration, and the growth situation and browning rate were recorded every 15 days. The concentration of kanamycin or hygromycin that inhibited callus proliferation was selected to screen resistant calli. Three independent repeats were performed.

### 2.4. Agrobacterium-Mediated Callus Transformation

*Agrobacterium tumefaciens* strain EHA105 harboring the pLGNe vector, which contains the β-glucuronidase gene (*GUS*) and kanamycin resistance gene (*NPTII*), was inoculated into a YEB liquid medium containing 50 mg L^−1^ rifampicin and 100 mg L^−1^ kanamycin and was cultured overnight at 28 °C with 200 rpm shaking. Then, the *A. tumefaciens* solution was centrifuged at 4000 rpm for 10 min and washed twice with liquid MS or a modified MS medium. The *A. tumefaciens* cells were gathered and diluted with different resuspensions A1 (MS + acetosyringone (AS) 100 μmol·L^−1^), A2 (MS without (NH_4_NO_3_) + AS 100 μmol·L^−1^), and A3 (MS without (KH_2_PO_4_, NH_4_NO_3_, KNO_3_, CaCl_2_) + AS 100 μmol·L^−1^) to OD600 concentrations of 0.2 and 0.4, respectively. Approximately 450 uniform calli (0.8 cm^3^) were infected for 5, 10 and 15 min and then cultured on the corresponding B1 (MS + 6-BA 1.0 mg L^−1^ + NAA 1.0 mg L^−1^ + AS 100 μmol·L^−1^), B2 (MS without (NH_4_NO_3_) + 6-BA 1.0 mg L^−1^ + NAA 1.0 mg L^−1^ + AS 100 μmol·L^−1^), and B3 (MS without (KH_2_PO_4_, NH_4_NO_3_, KNO_3_, CaCl_2_)+ 6-BA 1.0 mg L^−1^ + NAA 1.0 mg L^−1^ + AS 100 μmol·L^−1^) media in the dark for 3 days. After that, the infected callus was inoculated on MS medium for 3 days and checked for transient *GUS* expression and a *GUS*-positive rate (%) was defined as the *GUS*-stained callus number/callus infected number × 100%. In addition, the callus was infected by the EHA105 strain harboring the pCambia1301 vector using the optimal protocol before being transferred to the selection medium (callus proliferation medium with 300 mg·L^−1^ cefotaxime and 75 mg·L^−1^ hygromycin). At approximately three weeks, the surviving callus on the originally infected callus was transferred to a new selection medium. After three weeks, the newly grown callus on the selection medium was used for PCR identification and *GUS* staining, and three independent replicates were performed for transgenic frequency analysis.

### 2.5. GUS Staining and PCR Analysis of Transgenic Calli

The histochemical staining of GUS activity was performed according to the method described by Jefferson (1987) with some modifications. The non-transformed callus was used as a control, and the resistant callus was soaked in a staining solution containing 1 mg·mL^−1^ 5-bromo-4-chloro-3-indolylglucoside (X-Gluc) at 37 °C for 4 h. All calli were decolorized with 50% and 75% ethanol for 24 h in turn and were then observed under a microscope. Furthermore, the transformed callus and the control were used as the template for PCR analysis. The primers *GUS*-forwards (ATGTTACGTCCTGTAGAAACC) and *GUS*-reverse (GTGACGCACAGTTCATAGVG) were used to detect the insertion of exogenous *GUS* in transgenic calli. The PCR system was 20 μL, and the procedure was as follows: 95 °C for 5 min, then 38 cycles at 95 °C for 30 s, 58 °C for 45 s, 72 °C for 1 min, followed by 72 °C for 7 min. The PCR amplification products were identified by 1.2% gel electrophoresis.

### 2.6. Vector Construction and Transformation of PR Promoters

Based on the sequences of the *BjCHI1* promoter (AY714982), *ZmPR4* promoter (AJ969166), and plant binary expression vector pBI121, two pairs of specific primers, *BjCHI1*-forwards (ACCATGATTACGCCAAGCTTCCCATCCAAGAGTCCAAA) and *BjCHI1*-reverse (GACTGACCACCCGGGGATCCGTTTCTGAGCTGTATGGTTG) and *ZmPR4*-forwards (ACCATGATTACGCCAAGCTTTCGATGCTTCGCCGTATC) and *ZmPR4*-reverse (CCACCCGGGGATCCTCTAGAACGTATGTAGCTGCTACTTGC) were designed. Genomic DNA was extracted from mustard and maize leaves as a template, and the *BjCHI1* and *ZmPR4* promoters were amplified by PCR. The obtained PCR products were inserted into the binary vector pBI121 instead of the 35S promoter using the In-Fusion PCR Cloning System (NR005, Novoprotein, Suzhou, China). After sequencing, the resulting constructs were introduced into the EHA 105 strain and used to infect calli according to the optimum transgenic protocols.

### 2.7. Inducible GUS Activity Assay

The inducible medium containing *B. cinerea* filtrate was prepared based on the reported protocol [34] with some modifications. *B. cinerea* was cultured on a potato dextrose agar (PDA) medium at 28 °C for two weeks and then transferred to a 250 mL flask containing a 100 mL liquid PDA medium. The cultures were maintained at room temperature with continuous agitation at 100 rpm for one week and were subcultured by transferring 20 mL of the fungal culture to a 100 mL liquid PDA medium at one-week intervals. The cultures that had been subcultured at least twice were used for filtering. The filtrate was sterilized with a 0.22 μm filter unit and then added to the MS medium at a volume ratio of 1:10. After cocultivation for 3 d, approximately 30 transgenic calli were transferred to the induced medium containing *B. cinerea* filtrate and a basic MS medium for 48 h prior to *GUS* staining analysis. The GUS enzyme activity assay was based on the method of Jefferson (1987) [35]. The experiment was repeated at least three times.

### 2.8. Molecular Identification by Quantitative Real-Time PCR

Transgenic calli were identified at the genomic DNA level, which was carried out according to the PCR methods described in the section “2.6 *GUS* staining and PCR identification of transgenic calli”. To further analyze the transcription levels of the *GUS* gene, the total RNA was extracted from transgenic calli induced at 48 h and 0 h (as a control) using a TRIzol Reagent (15596026, Invitrogen, Waltham, MA, USA) and first-strand cDNA was synthesized from 1 μg RNA using a PrimeScript^TM^ RT Reagent Kit (RR047, TaKaRa, Otsu, Japan). A fluorometric quantitative RT–PCR was performed using the following program: 95 °C for 1 min, 40 cycles of 95 °C for 5 s, 60 °C for 30 s and 72 °C for 30 s in a 10 μL mixture containing 5 μL of SYBR Premix Ex Taq II (Tli RNaseH Plus) (RR820, TaKaRa), 1 μL of a 10-fold dilution of cDNA template, and specific primers *GUS*-QF (CGCCGATTGCGACCTCGC) and *GUS*-QR (CACCGAAGTTCATGCCAGTC). The lily 18S rRNA was used as a reference gene [31]. The relative expression of *GUS* genes was calculated by the 2^−ΔΔCT^ method [36]. The reactions were performed three times.

### 2.9. Data Statistics and Analysis

All experiments were performed with at least three replicates. The results are presented as the mean ± the standard error and were statistically analyzed using Excel 2020 (Microsoft Corporation, Redmond, WA, USA) and Statistical Product and Service Solutions 25.0 (SPSS Institute, IBM, Armonk, NY, USA). Duncan’s multiple comparison method or Student’s *t*-test was used to test for significant differences (*p* < 0.05).

## 3. Results

### 3.1. Induction and Proliferation of Embryogenic Calli

**Figure 1:**
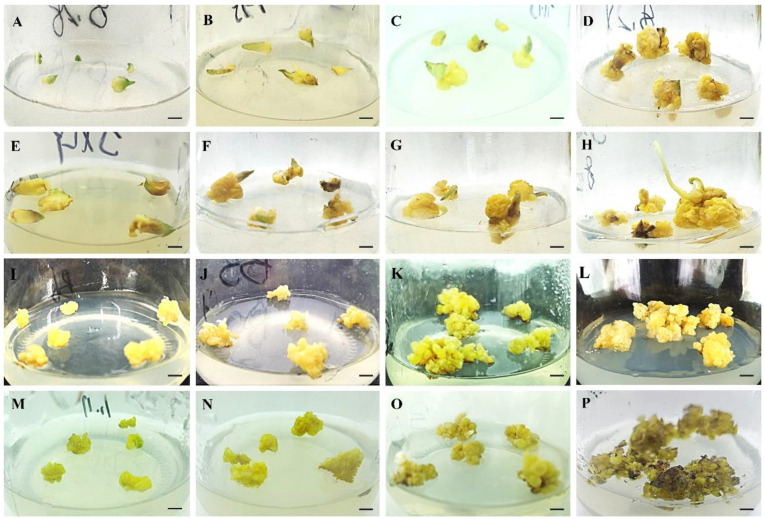
The callus induction and proliferation of different periods were recorded on the optimal medium. (**A**–**D**): Induced calli from bulblet scales on MS medium supplemented with 1.0 mg·L^−1^ PIC + 0.2 mg·L^−1^ NAA under darkness for 1–4 weeks; (**E**–**H**): Induced calli from bulblet scales on the optimum medium in the light for 1–4 weeks. (**I**–**L**): Proliferation callus on MS medium supplemented with 0.5 mg·L^−1^ 6-BA + 1.0 mg·L^−1^ NAA under darkness for 1–4 weeks; (**M**–**P**): Proliferation callus on the optimum medium in the light for 1–4 weeks. The bars represent 5 mm in length.

**Figure 2:**
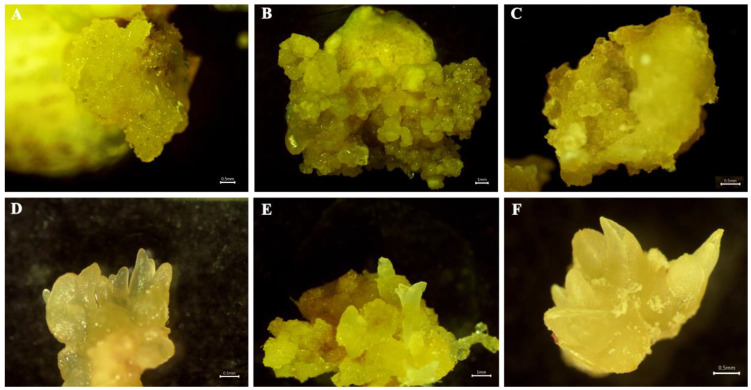
The induction and development of embryogenic calli was recorded in Longya lily. (**A**,**B**): Light yellow and granular embryogenic callus induced on the nonoptimum or the optimum medium for 30 days. (**C**): Considerable transparent particles in proliferation callus after culture with the optimum medium for 21 days. (**D**,**E**): The torpedo and cotyledonary stages of embryos after culture on the proliferation medium for 30 days. (**F**): The regenerated shoot on the proliferation medium for 45 days.

*In vitro* bulb scales were sterilized and cultured on an MS medium with 0.5 mg·L^−1^ 6-BA and 0.2 mg·L^−1^ NAA to generate small bulblets over approximately 6 weeks. The regenerated bulblet scales were inoculated on an MS medium with different concentrations of PIC and NAA in the dark to induce embryogenic calli. After 30 days, the results showed that MS medium supplemented with 1.0 mg·L^−1^ PIC + 0.2 mg·L^−1^ NAA induced a high quantity of embryogenic calli, and the induction rate was approximately 45.00% (Table 1). After 7 d of cultivation on the optimum medium, the scales were not changed. Until 14 d, the scales began to bend, and a few callus cells appeared on the surface. After cultivation for 21 d, a mass of callus emerged around the scales, and the callus amount increased significantly at 30 d, with slight compactness (Figure 1A–D). In contrast, the scales began to expand, and a few callus cells emerged after just 7 d of cultivation under light conditions (Figure 1E,H). Callus formation advanced significantly over time, and the frequency increased to 64.67%, suggesting that the light conditions contributed to callus induction. Meanwhile, granular, light yellow-colored, translucent somatic embryos were observed on the callus surface (Figure 2B).

To obtain as many embryogenic calli as possible, induced calli of the same size were subcultured on an MS medium with different concentrations of 6-BA and NAA in the dark. After 30 days, the results showed that the MS medium with combinations of 0.5 mg·L^−1^ 6-BA and 1.0 mg·L^−1^ NAA was predominant for callus growth, and the frequency of callus proliferation was 56.30% (Table S1). The callus growth status on the optimal medium was recorded at different periods (Figure 1I–L). There was almost no proliferation of calli at 7 d of culture, and the number of calli began to increase slowly from 7 to 14 d. Rapid proliferation occurred at approximately 14–21 d, after which the proliferation rate decreased, and a small browning callus could be observed at 30 d. During this step, we observed globular, torpedo and cotyledonary embryos (Figure 2C–F), which were involved in callus proliferation. Furthermore, the callus on the optimal medium was cultured under light conditions but did not proliferate after 7 d of culture, although some chlorophyll was detected. After that, the callus continued to slowly proliferate from 7 to 21 d, and rapid proliferation was observed at 21–30 d, while purple anthocyanins accumulated on the surface of the callus (Figure 1M–P). Thus, the optimum callus proliferation was cultivated on MS medium with 0.5 mg·L^−1^ 6-BA and 1.0 mg·L^−1^ NAA in darkness for one month, and the multiplication index reached 3.96 (Table S1).

### 3.2. Effects of Plant Growth Regulators on Shoot Induction and Proliferation

**Figure 3:**
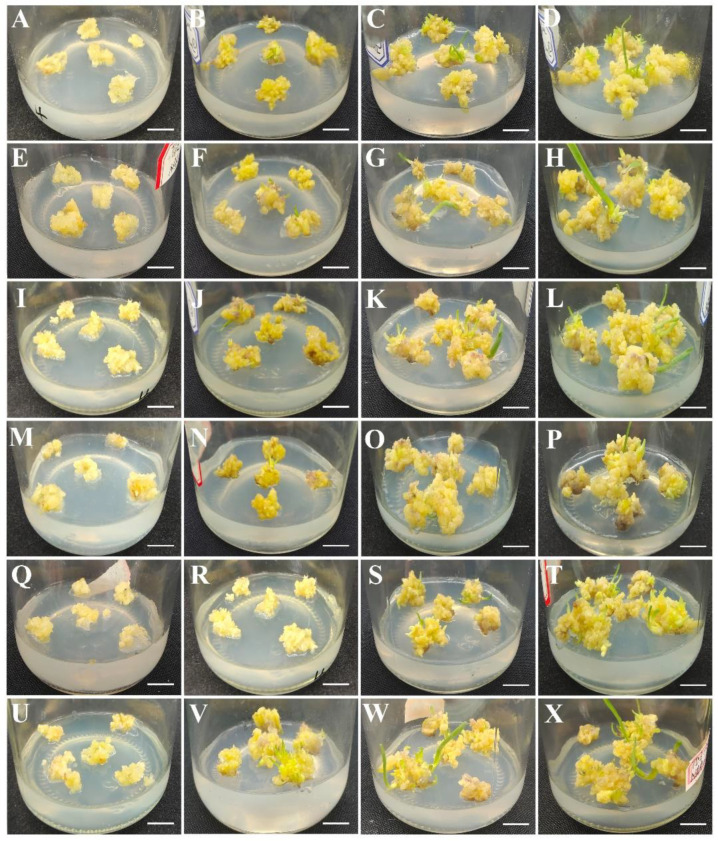
Shoot induction was performed on the media supplemented with different TDZ and NAA combinations. (**A**–**D**): The shoot induction on MS medium with 0.5 mg·L^−1^ TDZ and 0.2 mg·L^−1^ NAA for 0, 15, 30 and 45 days. (**E**–**H**): The shoot induction on MS medium with 0.5 mg·L^−1^ TDZ and 0.4 mg·L^−1^ NAA for 0, 15, 30 and 45 days. (**I**–**L**): The shoot induction on MS medium with 1.0 mg·L^−1^ TDZ and 0.2 mg·L^−1^ NAA for 0, 15, 30 and 45 days. (**M**–**P**): The shoot induction on MS medium with 1.0 mg·L^−1^ TDZ and 0.4 mg·L^−1^ NAA for 0, 15, 30 and 45 days. (**Q**–**T**): The shoot induction on MS medium with 2.0 mg·L^−1^ TDZ and 0.2 mg·L^−1^ NAA for 0, 15, 30 and 45 days. (**U**–**X**): the shoot induction on MS medium with 2.0 mg·L^−1^ TDZ and 0.4 mg·L^−1^ NAA for 0, 15, 30 and 45 days. Bars represent 1 cm.

Different concentrations of plant growth regulators (PGRs), including TDZ and NAA, were added to an MS basic medium for the maturation and germination of embryos. After 15 days of culture, some shoots began to appear on the callus surface. More shoots grew, and their length also gradually increased from 15 to 30 days. At the same time, the calli obviously proliferated. At 45 days, the shoots were significantly induced and associated with a few slightly browning calli (Figure 3). The data showed that an MS medium with 2.0 mg·L^−1^ TDZ and 0.4 mg·L^−1^ NAA was the optimum for shoot induction, with a 92.22% callus shooting rate and a 3.91% shooting coefficient (Table 2).

**Figure 4:**
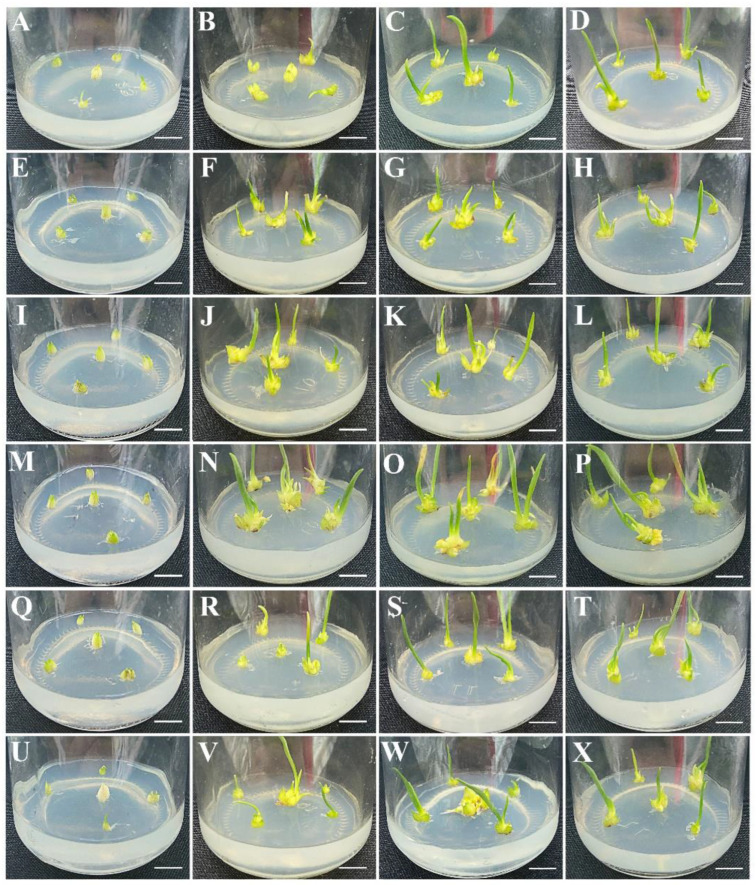
Shoot proliferation was performed on media supplemented with different TDZ and NAA combinations. (**A**–**D**): Shoot proliferation on MS medium with 0.5 mg·L^−1^ TDZ and 0.2 mg·L^−1^ NAA for 0, 15, 30 and 45 days. (**E**–**H**): Shoot proliferation on MS medium with 0.5 mg·L^−1^ TDZ and 0.5 mg·L^−1^ NAA for 0, 15, 30 and 45 days. (**I**–**L**): Shoot proliferation on MS medium with 1.0 mg·L^−1^ TDZ and 0.2 mg·L^−1^ NAA for 0, 15, 30 and 45 days. (**M**–**P**): Shoot proliferation on MS medium with 1.0 mg·L^−1^ TDZ and 0.5 mg·L^−1^ NAA for 0, 15, 30 and 45 days. (**Q**–**T**): Shoot proliferation on MS medium with 2.0 mg·L^−1^ TDZ and 0.2 mg·L^−1^ NAA for 0, 15, 30 and 45 days. (**U**–**X**): Shoot proliferation on MS medium with 2.0 mg·L^−1^ TDZ and 0.5 mg·L^−1^ NAA for 0, 15, 30 and 45 days. Bars represent 1 cm.

To obtain a better shoot proliferation, slim shoots from the induction medium were transferred into a proliferation MS medium with different TDZ and NAA PGR combinations. The shoots started to proliferate after approximately 15 days of culture, after which many more shoots developed, and the length of the shoots obviously increased after 30 days. From 30 to 45 days, shoot proliferation slowed, while the shoots became much longer than before (Figure 4). An analysis of the results showed that the MS medium containing 1.0 mg·L^−1^ TDZ and 0.5 mg·L^−1^ NAA mostly promoted shoot proliferation. The rate of shoot proliferation was 96.67, and the proliferation coefficient reached 3.28 (Table S2).

### 3.3. Effects of Macroelements and PGRs on Shoot Rooting Induction

**Figure 5:**
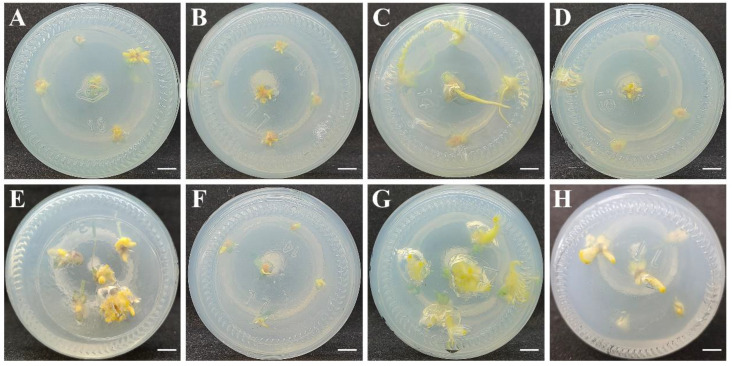
Shoot rooting on the different combinations of media for 30 days. (**A**,**E**): Shoot rooting on 1/2 MS(A) or MS(E) with 0.5 mg·L^−1^ NAA and 0.2 mg·L^−1^ 6-BA. (**B**,**F**): Shoot rooting on 1/2 MS(B) or MS(F) with 0.2 mg·L^−1^ NAA and 0.5 mg·L^−1^ 6-BA. (**C**,**G**): Shoot rooting on 1/2 MS(C) or MS(G) with 0.3 mg·L^−1^ NAA. (**D**,**H**): Shoot rooting on 1/2 MS(D) or MS(H) with 0.3 mg·L^−1^ 6-BA. Bars represent 1 cm.

Considering that root induction is generally regulated by macroelements and PGRs, including 6-BA and NAA, eight combinations constructed by the MS or 1/2 MS medium with different concentrations of 6-BA and NAA were used to determine the optimal conditions for induction of shoot rooting. The results showed that roots were directly induced from a mixture of shoots (Figure S1) and clearly observed in the eight combinations after 30 days of culture (Figure 5). Further quantitative analysis revealed that the maximal rooting rate was 99% and that the rooting coefficient was 5.34 (Table 3). Therefore, an MS medium containing 0.3 mg·L^−1^ of NAA was suitable for the induction of shoot rooting in Longya lilies.

### 3.4. Sensitivity Test of the Callus to Antibiotics

**Figure 6:**
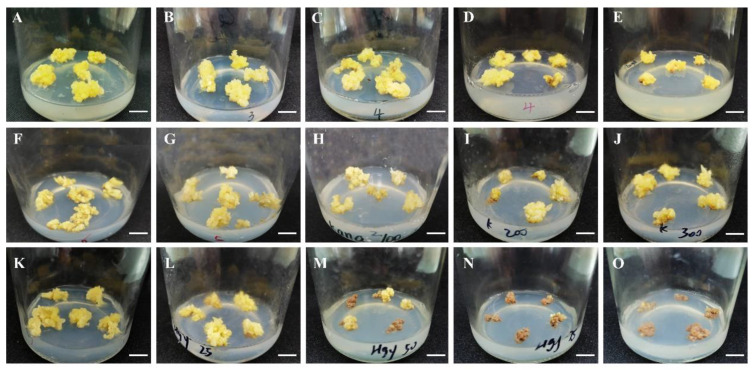
Effects of different concentrations of antibiotics on callus browning. (**A**–**E**): The growth status of calli on an optimum proliferation medium with 0 (**A**), 100 (**B**), 200 (**C**), 300 (**D**), and 400 mg·L^−1^ (**E**) cefotaxime for 30 days; (**F**–**J**): tThe growth status of calli on the optimum proliferation medium with 0 (**F**), 50 (**G**), 100 (**H**), 200 (**I**), and 300 mg·L^−1^ (**J**) kanamycin for 30 days; (**K**–**O**): The growth status of calli on optimum proliferation medium with 0 (**K**), 25 (**L**), 50 (**M**), 75 (**N**), and 100 mg·L^−1^ (**O**) hygromycin for 30 days. The bars represent the length of 10 mm.

To select the optimum concentration of antibiotics for screening transgenic calli, uniform calli were cultivated in an MS proliferation medium containing different concentrations of cefotaxime (Cef), kanamycin (Kan) and hygromycin (Hyg). The effects of antibiotics on callus growth were observed after 30 d of cultivation in the dark (Table S3 and Figure 6). Almost all of the calli grew well and rapidly proliferated in the control (without any antibiotics). Meanwhile, the calli were less brown and proliferated normally on a medium supplemented with 100 or 200 mg·L^−1^ Cef, and there was little browning and decreased proliferation with 300 or 400 mg·L^−1^ Cef. In addition, the callus was not sensitive to Kan at concentrations lower than 200 mg·L^−1^ and displayed partial browning (32.67%) and decreased proliferation when the concentration of Kan increased to 300 mg·L^−1^. However, the callus decreased in its proliferation with a 46.67% browning rate when 25 mg·L^−1^ Hyg was added to the medium. With a further increase in the Hyg concentration to 50 mg·L^−1^, the proliferation was slow and slight, and more calli changed to brown (75.56%). When the concentration was as high as 75 mg·L^−1^, most of the calli (96.67%) were browning, and no proliferation was observed. There were few calli alive in the medium with 100 mg·L^−1^ Hyg, in which callus differentiation and proliferation were completely inhibited. A high selection pressure contributed to obtaining positive transformants but repressed shoot induction and development; thus, the combination of 300 mg·L^−1^ Cef and 75 mg·L^−1^ Hyg was selected for screening transgenic resistant calli.

### 3.5. Transformation System and Obtaining Transgenic Calli

**Figure 7:**
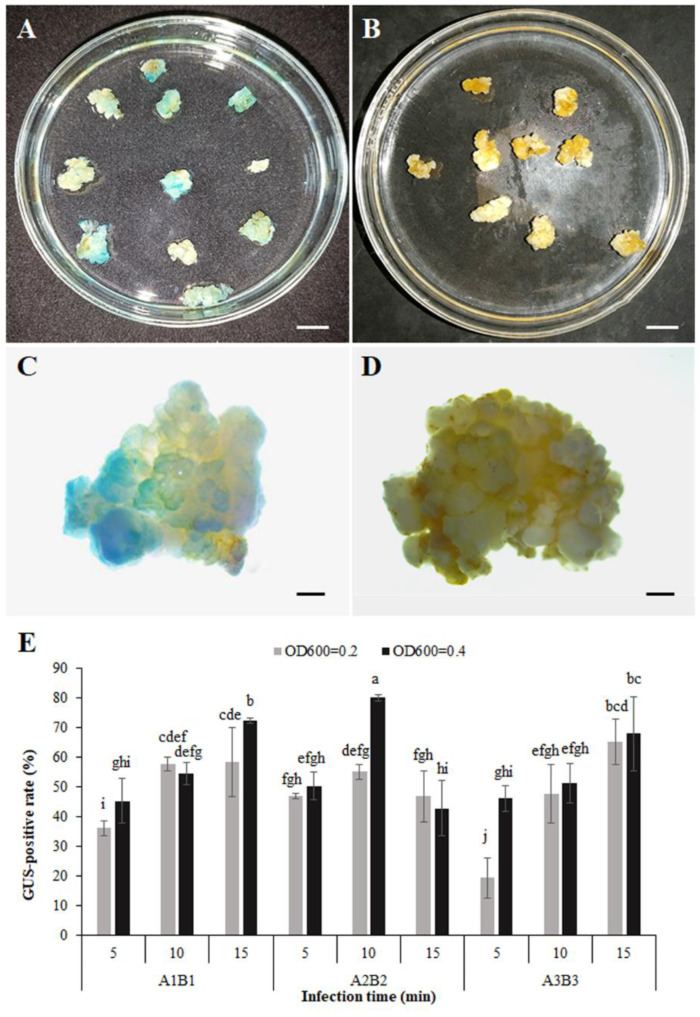
Histochemical staining detection of *GUS* gene expression in transformed calli. (**A**,**C**): Observation of *GUS* gene expression in transformed calli directly and under stereoscopes. (**B**,**D**): Observation of non-transformed calli directly and under stereoscopes. (**E**): Statistical analysis of *Agrobacterium*-mediated transformation in embryogenic calli by Duncan’s test. Different letters indicate significant differences by Duncan’s test (*p* < 0.05). The scale bars represent 8 mm (**A**,**B**), and 1 mm (**C**,**D**).

To construct a highly effective transformation method, *A. tumefaciens* strain EHA105 harboring the pLGNe vector containing the *GUS* reporter gene was used to infect the callus. A series of combinations of suspension solution and coculture medium (A1B1, A2B2, A3B3), with suspension concentration at OD_600_ (0.2 and 0.4) and infection time (5, 10, and 15 min) were used to select the optimal transformation protocols. As shown in Figure 7, the *GUS*-positive rate increased with increasing infection time at the same suspension concentration using MS (A1B1) or modified MS (A3B3) as the suspension solution and coculture medium. The *GUS*-positive rate at an infection time of 15 min was lower than that at infection times of 5 and 10 min using the modified MS (A2B2) as the suspension solution and coculture medium at OD_600_ = 0.4. In addition, a higher *GUS*-positive rate was observed at higher concentrations (OD_600_ = 0.4) than at lower concentrations (OD_600_ = 0.2) in most cases. Compared with the basic MS (A1B1), the highest *GUS*-positive rate (80.19%) occurred with modified MS (A2B2) at a concentration of OD_600_ = 0.4 infected with 10 min. However, the *GUS*-positive rate (67.94%) in the modified MS (A3B3) was similar to that in the basic MS (A1B1). Therefore, *Agrobacterium* with a concentration of 0.4 at OD_600_ using the modified MS (A2B2) infected for 10 min was suitable for improving the transformation frequency.

**Figure 8:**
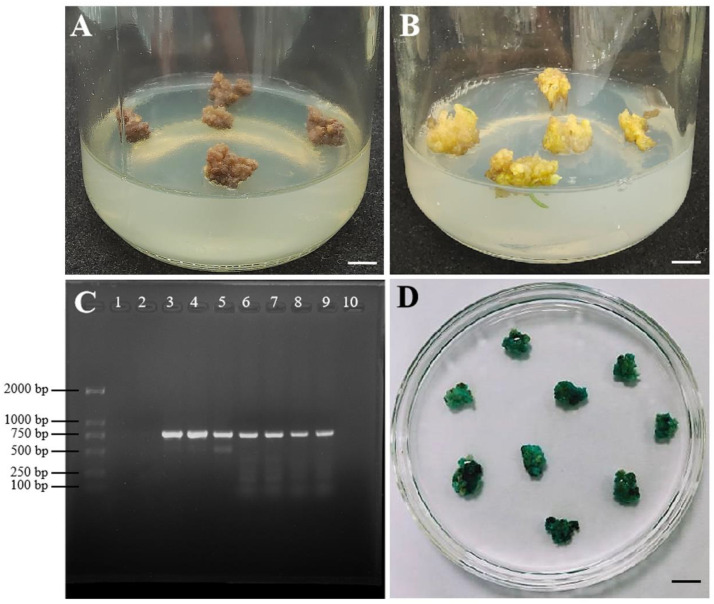
Identification of the resistant callus by *GUS* staining and PCR checked methods. (**A**,**B**): The non-transformed callus (**A**) and transformed callus (**B**) cultured on the selected medium for 3 weeks. New growth calli were observed from the transformed calli. (**C**): PCR analysis of the *GUS* gene in transgenic calli. M: Marker 2000; 1: blank control (ddH_2_O); 2: nontransgenic callus. 3–9: *GUS*-positive callus. (**D**): *GUS* staining of resistant calli. The bar represents 6 mm (**A**,**B**) and 9 mm (**D**) in length.

In addition, the infected callus was cultivated for approximately three weeks (Figure 8A,B) on the selection medium. The new callus was separated from the original callus and transferred to a new selection medium. After proliferation for another three weeks, the resistant callus was randomly selected for a *GUS* histochemical staining check. Compared with the control, all the selected resistant calli were stained blue (Figure 8D), suggesting that this plasmid containing the *GUS* reporter gene was transferred into the callus. To further identify transgenic events, PCR amplification was used to analyze the insertion of the *GUS* gene in the callus at the molecular level. The results (Figure 8C) showed that *GUS* stripes were clearly observed in transgenic calli but were not detected in nontransformed calli. The data showed that the transgenic frequency reached an average of 65.56% in three independent tests. To further verify the transformation events, 38 transgenic seedlings grown on selected media for 30 days were used for GUS staining, and about 28 transgenic seedlings became blue in color in relation to the blank control (Figure S2)

### 3.6. Expression Responses to B. cinerea of PR Promoter-GUS in Transgenic Calli 

**Figure 9:**
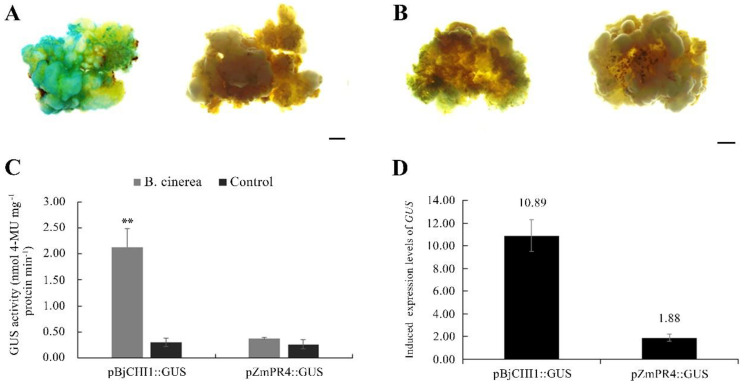
Expression responses of the PR promoter *BjCHI1* or *ZmPR4* to *B. cinerea* in transgenic calli. (**A**): The *GUS* staining analysis of transgenic calli containing the *BjCHI1* promoter−*GUS* on a medium with *B. cinerea* filtrate (left) and without *B. cinerea* filtrate (right); (**B**): *GUS* staining of transgenic calli containing *ZmPR4* promoter−*GUS* on a medium with *B. cinerea* filtrate (left) and without *B. cinerea* filtrate (right); (**C**): Quantitative determination of GUS activity in transgenic calli containing *BjCHI1* promoter−*GUS* or *ZmPR4* promoter−*GUS*. The bars represent the length of 1 mm (**A**,**B**) and the stars (**) in C represent significant differences (*t* test, *p* < 0.01). (**D**): qRT−PCR analysis of *GUS* transcriptional levels driven by *BjCHI1* and *ZmPR4* promoters in transgenic calli cultivated on a medium with or without *B. cinerea* filtrate.

To analyze the expression response of PR promoters in Longya lily to *B. cinerea*, two *GUS* fusion expression vectors driven by *BjCHI1* and *ZmPR4* promoters were constructed. *BjCHI1* and *ZmPR4* promoters were amplified from the mustard and maize B73 genome DNA and cloned into the PBI121 vector located in the frontier of the *GUS* gene. After digestion by restriction endonucleases (Figure S2), both vectors were verified by sequencing analysis. The *BjCHI1* and *ZmPR4* promoters displayed 99.54% and 100% consistency with the original sequences (AY714982.1 and AJ969166.1, respectively). The *A. tumefaciens* strain EHA105 containing a recombinant *BjCHI1* promoter-*GUS,* or *ZmPR4* promoter-*GUS* vector, was used to infect lily calli using the optimal transformation protocol. *GUS* staining indicated that transgenic calli containing *BjCHI1* promoter-*GUS* or *ZmPR4* promoter-*GUS* were not colored on the control medium; however, transgenic calli containing *BjCHI1* promoter-*GUS* were clearly blue, and those containing *ZmPR4* promoter-*GUS* were not significantly blue when cultivated on a medium with a *B. cinerea* solution (Figure 9A,B). The induced efficiency of the *GUS* expression reached 66.40% on average (Table S4). Moreover, GUS activity analysis showed similar results to the *GUS* staining results (Figure 9C).

To confirm transgenic calli at the molecular level, calli were randomly selected to detect the insertion of the *GUS* gene driven by *BjCHI1* and *ZmPR4* promoters using PCR methods. The results showed that the *GUS* gene could not be found in non-transformed calli, while clear bands of *GUS* were observed in all transgenic calli containing the *BjCHI1* promoter-*GUS* or *ZmPR4* promoter-*GUS* (Figure S3). A further qRT–PCR analysis showed that compared to the control, the *GUS* gene driven by the *BjCHI1* promoter was significantly upregulated when transgenic calli containing the *BjCHI1* promoter-*GUS* were cultured on a medium supplemented with *B. cinerea* solution. The *GUS* gene driven by the *ZmPR4* promoter showed similar transcription levels when transgenic calli containing *ZmPR4* promoter-*GUS* were cultured on a medium with or without *B. cinerea* solution (Figure 9D).

## 4. Discussion

Cohen and Meredith (1992) [37] and Langeveld et al. (1995) [38] found that *Agrobacterium* could infect lilies (*Lilium* spp.); from this, efficient plant regeneration and genetic transformation systems have been established via *Agrobacterium tumefaciens* in different lily species and varieties, such as *L. longiflorum* Thunb., *L. pumilum* DC., *L. davidii* var. *unicolour*, *L. lancifolium* Thunb., Oriental hybrids ‘Siberia’, ‘Sorbonne’, ‘Acapulco’, Asiatic ‘Elite’, *L. longiflorum* ‘White Heaven’ and Oriental × Trumpet ‘Robina’ [12,13,31,39]. The Longya lily is an important source of traditional Chinese medicine derived from lilies, but the *Botrytis* fungal disease seriously affects its yield and quality [2]. There have been very few reports on the disease resistance-related improvement of Longya lily until now. Compared with particle bombardment transformation, *Agrobacterium*-mediated transformation is an efficient and low-cost system that has been widely used for stable gene transfer. Therefore, in this study, we performed a highly efficient plant regeneration and callus genetic transformation using an *Agrobacterium*-mediated method in the Longya lily. Moreover, the expression responses of two pathogen-inducible PR promoters (*ZmPR4* and *BjCHI1*) to *B. cinerea* were identified in transgenic calli.

In previous studies, embryogenic calli were considered the ideal receptor for plant regeneration and highly efficient, reliable and stable genetic transformation [11,12,13,40,41]. In addition, two factors, PGRs and light conditions, have been found to play key roles in inducing lily callus formation, and the optimum conditions were shown to be distinct for callus induction in different lilies or for selecting different explants in the same lily [42,43,44,45,46]. In our results, an MS medium with 1.0 mg L^−1^ PIC and 0.2 mg L^−1^ NAA was appropriate for embryogenic callus induction, which was consistent with the concentration of PGR combinations that have been used to induce the embryogenic calli of *L. pumilum* DC. Fisch., although the induced frequency (64.67%) in the Longya lily was less than this (90.7%) in *L. pumilum* [11]. The frequency induced by the combination of PIC and NAA for *L. brownie* var. *viridulum* was higher than what has previously been found (57.14%) using the combinations of 1 mg·L^−1^ 2,4-D, 0.3 mg·L^−1^ NAA and 0.5 mg·L^−1^ KT [47]. Otherwise, we observed that under light conditions, the frequency of inducing embryogenic calli was much higher (64.67%) than that in the dark (45.00%) and was similar to that in *L. leucanthum* (Baker) Baker and *L. brownii* var. *viridulum* [44,47]. These findings suggest that light might contribute to the induction of embryogenic calli.

Lily calli can be subcultured with an inducing medium to achieve proliferation [45,48,49]. In an early test by our group, a combination of PIC and NAA was used to induce callus proliferation in the Longya lily, but the result was not satisfactory. An MS medium with a combination of 6-BA and NAA was found to effectively promote the growth of embryogenic calli [50]. Herein, an MS medium with 0.5 mg·L^−1^ 6-BA and 1.0 mg·L^−1^ NAA induced rapid callus proliferation. However, the calli were accumulated with a small amount of chlorophyll and many anthocyanins under light conditions, which was different from the callus in *L. brownii* var. *giganteum* and *L*. *martagon* [50,51], suggesting that anthocyanins were easily induced in Longya lily via light stimulation. Therefore, the callus was chosen for the following experiment in darkness because anthocyanins affect the callus quality, even though the callus proliferated more efficiently under light conditions (coefficient of 5.19) than in darkness (coefficient of 3.96).

Shoot differentiation is a bottleneck step for highly effective plant regeneration and genetic transformation. Some reports have shown that the combination of auxin and cytokinin can be employed for the differentiation or redifferentiation of explants in tissue cultures [52]. In the propagation of the Longya lily, the combination of 6-BA and NAA or KT and NAA was suitable for shoot induction and proliferation from bulb scales [53,54]. TDZ, as a phenylurea compound, has high cytokinin activity, which can induce adventitious shoot organogenesis [55,56]. Herein, different combinations of TDZ and NAA were first employed for shoot induction and the proliferation of Longya lily. The shooting rate (92.22%) and shooting efficiency per callus (3.91) were greater than those in *L. martagon* [50] and in the *Longiflorum* × *Asiatic* (LA) hybrid ‘Eyeliner’ [57]. By contrast, the shoot proliferation (coefficient of 3.28) in Longya lily was lower than that (4.5) in *L. pumilum* [11] and (4.69) in the LA hybrid ‘Eyeliner’ [57]. In addition, the rooting rate in our study reached 99.00% after 30 days of culture, which was superior to that (87% and 93.33%) in previous studies [53,54].

In *Agrobacterium*-mediated genetic transformation, antibiotics such as kanamycin and hygromycin are important for screening transgenic positive seedlings. The optimal concentrations of kanamycin (ranging from 50 to 200 mg·L^−1^) and hygromycin (ranging from 7.5 to 75 mg·L^−1^) have been used to select regenerated resistant plantlets in different lily species and varieties [12,26,27,30,58]. In the same lily, such as in *L. longiflorum* ‘White-elegance’, the oriental lily ‘Siberia’ and ‘Sorbonne’, an appropriate concentration of kanamycin was also variable, from 75 to 120 mg·L^−1^ for different explant materials, such as scale leaves, bulblets, and petioles [58]. In our study, the calli were sensitive to 75 or 100 mg·L^−1^ hygromycin, which could be used for screening resistant calli. However, a high selection pressure was applied against shoot induction and growth; therefore, 75 mg·L^−1^ hygromycin was more advantageous in the selection. In addition, the callus browning rate was only 32.67% when 300 mg·L^−1^ kanamycin was used, suggesting that kanamycin was unsuited for selection, similar to what was observed in *L. brownii* var. *giganteum* and *L. longiflorum* ‘Nellie White’ [59,60]. For cefotaxime, 200 mg·L^−1^ was previously added to inhibit the growth of *Agrobacterium* in the test but could not inhibit bacterial growth thoroughly. Thus, 300 mg·L^−1^ cefotaxime was used in the selection, similar to that used for *L. pumilum* DC. [12].

The explants, bacterial concentration, infection time, components of the suspension and coculture medium are important factors influencing transformation efficiency in lilies [8,26,27,31,61]. For example, increasing the *Agrobacterium* concentration or infection time has been shown to facilitate transformation efficiency [8,61]. Based on our results, in most cases, the transformation efficiency of *Agrobacterium* at an OD_600_ of 0.4 was higher than that at an OD_600_ of 0.2. When MS (A1B1) and modified MS (A3B3) were used as the suspension solution and coculture medium, the longer the infection time was, the higher the efficiency was. Ultimately, we obtained the highest efficiency using a modified MS (A2B2) to remove NH_4_NO_3_ by optimizing the infection time and bacterial concentration. This result was consistent with previous findings that decreased the concentration of NH_4_NO_3_ and generated a significant increase in the *GUS*-positive rate [26]. However, this transformation efficiency was not significantly increased in modified MS (A3B3) after removing four macroelements, which was different from the result in *L. formolongi* [27]. The *GUS* transient transformation rate (80.19%) was slightly higher than that in the *L. pumilum* DC, and the resistant callus rate (65.56%) was similar to that in *L. pumilum* DC [12]. Some GUS-blue transgenic seedlings further validated calli transformation for the Longya lily, similar to that in *L. pumilum* DC. and *L. longiflorum* [12].

The pathogenesis-related protein (PR) promoter is a pathogen-inducible promoter that has potential applications in the disease-resistance molecular breeding of plants [62,63]. PR promoters of maize *ZmPR4* and mustard *BjCHI1* containing cis-acting elements respond to pathogen infection. The *ZmPR4* promoter has been shown to be induced by the blast fungus *Magnaporthe grisea* in transgenic rice or *Rhizoctonia cerealis* in wheat embryonic calli [22,23], and the *BjCHI1* promoter has been induced in *Arabidopsis* and tobacco leaves by *B. cinerea* [25]. Here, using the *GUS* gene as a reporter gene, we tested the transcription levels of the *GUS* and GUS enzyme activity driven by both *ZmPR4* and *BjCHI1* promoters in transgenic calli. Our results revealed the low background expression of both promoters, but only the *BjCHI1* promoter was highly inducible in *B. cinerea*, which is probably related to the six w-box-like elements involved in the promoter [25]. The responsive level of *GUS* expression driven by the *BjCHI1* promoter in the transgenic calli was similar to that of the PPP1 promoter in response to *Xanthomonas axonopodis* pv. *citri* inoculation and was slightly lower than that of PPP3 in response to *Ralstonia solanaceous* infection [21,64]. This analysis provided a *B. cinerea*-responsive *BjCHI1* promoter, which could be used for the disease-resistance improvement of the Longya lily in future work.

## 5. Conclusions

In this study, embryogenic calli were induced using bulblet scales as explants, and a highly efficient regeneration system was successfully constructed for the Longya lily. The combination of 300 mg·L^−1^ Cef and 75 mg·L^−1^ Hyg was suitable for screening resistant calli, and *Agrobacterium*-mediated transformation was established with transgenic frequency (65.56%) in the calli of Longya lily. The PR promoter *BjCHI1* but not *ZmPR4* positively responded to *B. cinerea* in transgenic calli. Our research lays a foundation for obtaining transgenic lily plants and improving the disease resistance of the Longya lily.

## Figures and Tables

**Table 1:** Effects of picloram (PIC) and 1-naphthaleneacetic acid (NAA) combinations on the induction of embryogenic calli.

MS/(mg·L^−1^)		Scale Number	Number of Scales Inducing Embryogenic Callus	Frequency of Scales Inducing Embryogenic Callus (%)
PIC	NAA			
0.5	0.2	58.33 ± 2.19 a	10.00 ± 2.00 c	17.28 ± 3.10 de
0.5	0.5	50.67 ± 9.33 a	6.33 ± 2.85 c	12.78 ± 4.34 e
1.0	0.2	51.67 ± 10.93 a	22.67 ± 3.84 b	45.00 ± 2.89 b
1.0	0.5	47.00 ± 8.50 a	14.67 ± 3.28 bc	31.08 ± 2.66 c
2.0	0.2	48.00 ± 7.57 a	9.67 ± 2.73 c	23.56 ± 1.09 cd
2.0	0.5	49.00 ± 6.66 a	8.00 ± 1.53 c	16.01 ± 1.30 de
* 1.0	0.2	50.00 ± 0.00 a	32.33 ± 1.67 a	64.67 ± 3.33 a

Note: The stars represent the induced callus in the lightness. The data in the table show the mean ± standard error. Different letters behind each column indicate significant differences by Duncan’s test (*p* < 0.05). The same below.

**Table 2:** Effects of different thidiazuron (TDZ) and 1-naphthaleneacetic acid (NAA) combinations on shoot induction.

MS/(mg·L^−1^)		Callus Number	Number of Shooting Callus	Total Shooting Number	Callus Shooting Rate (%)	Callus Shooting Coefficient	Shoots Features	
TDZ NAA							Texture	Colour
0.5	0.2	30.00	25.33 ± 1.09 ab	85.33 ± 9.98 b	84.44 ± 3.64 ab	2.84 ± 0.33 b	Most short, less long	Tender green or light-yellow
0.5	0.4	30.00	22.67 ± 1.36 b	68.00 ± 7.76 b	75.55 ± 4.55 b	2.27 ± 0.26 b	Most short, less long	Light-yellow
1.0	0.2	30.00	25.00 ± 1.53 ab	77.00 ± 10.44 b	83.33 ± 5.09 ab	2.56 ± 0.35 b	All short	Tender green or green
1.0	0.4	30.00	24.67 ± 1.42 ab	71.00 ± 8.28 b	82.22 ± 4.75 ab	2.37 ± 0.28 b	All short	Tender green or green
2.0	0.2	30.00	25.33 ± 1.67 ab	68.33 ± 8.38 b	84.45 ± 3.88 ab	2.28 ± 0.28 b	All short	Tender green or light-yellow
2.0	0.4	30.00	25.11 ± 0.52 a	117.33 ± 13.50 a	92.22 ± 2.42 a	3.91 ± 0.45 a	Most short, less long	Tender green or green

**Table 3:** Effects of different combinations of macroelements, 6-benzylaminopurine (6-BA) and 1-naphthaleneacetic acid (NAA) on shoot rooting.

Media/(mg·L^−1^)	Inoculated Clustered Shoots	Number of Rooting Shoots	Total Induced Roots Number	Root Induction Rate (%)	Rooting Coefficient	Root Features	
						Texture	Color
**1/2 MS + 0.5 NAA + 0.2 6-BA**	30	29.00 ± 0.58 a	107.67 ± 14.94 b	96.67 ± 2.03 a	3.70 ± 0.46 b	A few, sturdy	Yellow
**1/2 MS + 0.2 NAA + 0.5 6-BA**	30	23.00 ± 1.53 b	82.00 ± 8.54 c	76.67 ± 4.91 b	3.58 ± 0.33 b	A few, slim	Light yellow
**1/2 MS + 0.3 6-BA**	30	21.33 ± 0.67 b	32.00 ± 4.16 e	71.00 ± 2.00 b	1.49 ± 0.16 d	A few, sturdy	Yellow
**1/2 MS + 0.3 NAA**	30	28.67 ± 0.88 a	110.67 ± 9.13 b	95.67 ± 2.96 a	3.85 ± 0.21 b	A few, slim	Light yellow
**MS + 0.5 NAA + 0.2 6-BA**	30	28.33 ± 0.67 a	116.67 ± 5.55 b	94.67 ± 2.33 a	4.12 ± 0.13 b	Much, sturdy	Yellow
**MS + 0.2 NAA + 0.5 6-BA**	30	22.67 ± 0.88 b	61.67 ± 5.23 cd	75.67 ± 2.96 b	2.74 ± 0.28 c	A few, slim	Light yellow
**MS + 0.3 NAA**	30	29.67 ± 0.33 a	158.67 ± 9.60 a	99.00 ± 1.00 a	5.34 ± 0.27 a	Much, sturdy	Yellow
**MS + 0.3 6-BA**	30	23.33 ± 0.88 b	48.00 ± 3.46 de	77.67 ± 2.91 b	2.05 ± 0.07 cd	A few, sturdy	Light yellow

## Data Availability

All supporting data can be found as an additional file with the manuscript.

## Supplementary Materials

The following supporting information can be downloaded at: https://www.mdpi.com/article/10.3390/plants12101992/s1, Table S1. Effects of 6-BA and NAA combinations on the proliferation of embryogenic calli (30 d). Table S2. Effects of different TDZ and NAA combinations on the shoot proliferation (45 d). Table S3. Effects of different antibiotic concentrations on the callus browning (30 d). Table S4. Summary of the induced expression of the *GUS* gene. Figure S1. Rooting shoots on the different combinations of media for 30 days. Figure S2. Representative transgenic seedlings and the GUS staining analysis. Figure S3. Analysis of recombinant *BjCHI1*::*GUS* and *ZmPR4*::*GUS* plasmids by digestion reaction. Figure S4. PCR analysis of the transformation of *BjCHI1*/*ZmPR4*::*GUS* in transgenic calli.
