# Supplementary material for: Establishment of Highly Efficient Plant Regeneration, Callus Transformation and Analysis of Botrytis cinerea-Responsive PR Promoters in Lilium brownii var. viridulum
Source: Plants (Basel). 2023 May 16;12(10):1992. doi: 10.3390/plants12101992 (PMC10221712; doi:10.3390/plants12101992)
Supplement: Supplementary file 1 [file plants-12-01992-s001.zip › plants-2298569-supplementary figures.pdf]

Supplementary Figures:

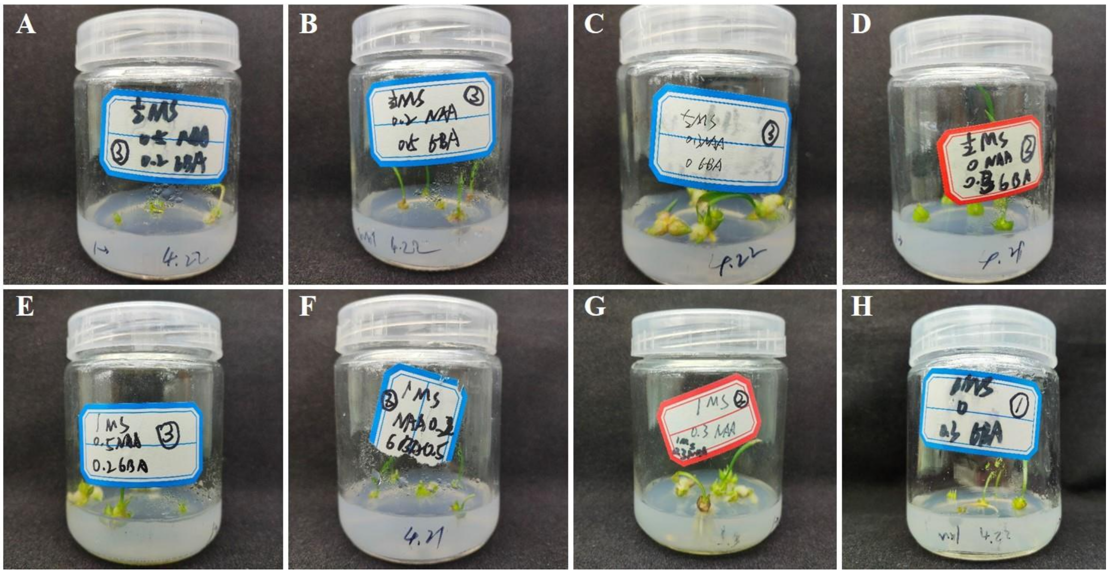

Figure S1. Rooting shoots on the different combinations of media for 30 days.

A, E: the shoot rooting on 1/2 MS(A) or MS(E) with 0.5 mg·L^{-1} NAA and 0.2 mg·L^{-1} 6-BA; B, F: the shoot rooting on 1/2 MS(B) or MS(F) with 0.2 mg·L^{-1} NAA and 0.5 mg·L^{-1} 6-BA; C, G: the shoot rooting on 1/2 MS(C) or MS(G) with 0.3 mg·L^{-1}mg/L NAA; D, H: the shoot rooting on 1/2MS(D) or MS(H) with 0.3 mg·L^{-1}mg/L 6-BA. The diameter of bottles was 6 cm.

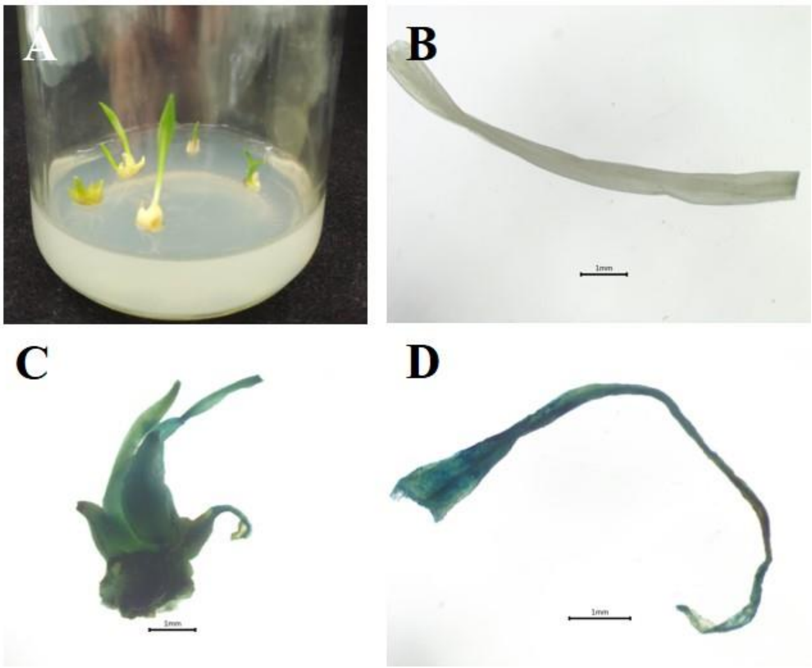

Figure S2. Representative transgenic seedlings and the GUS staining analysis.

A: The transformed seedlings grown on the selected medium for 30 days. The diameter of bottles was 6 cm. B: GUS staining of the leaf in non-transformed seedlings as the control; C,D: GUS staining of transformed seedlings including the bulblet scales (C) and the leaf (D). The bars represents 1 mm.

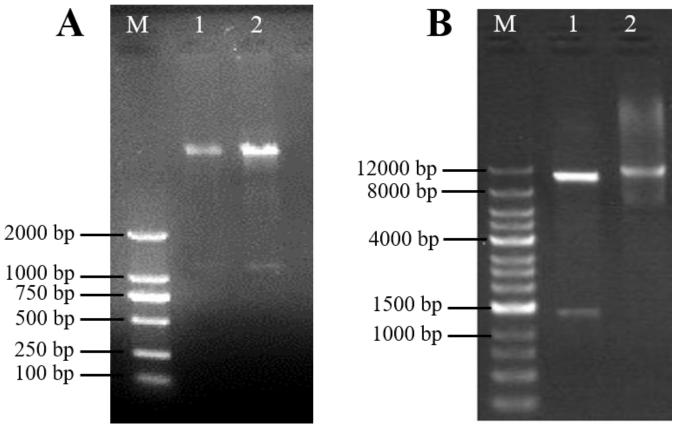

Figure S3. Analysis of recombinant BjCHI1::*GUS* and ZmPR4::*GUS* plasmids by digestion reaction.

A: The BjCHI1::GUS plasmid digested by Hind III and Xba I, M: Marker 2000; 1, 2: BjCHI1::GUS fragments digestion by Hind III and Xba I; B: The ZmPR4::GUS plasmid digested by Hind III and BamH I, M: 1 Kb ladder; 1: ZmPR4::GUS fragments digestion by Hind III and BamH I; 2: the plasmid ZmPR4::GUS.

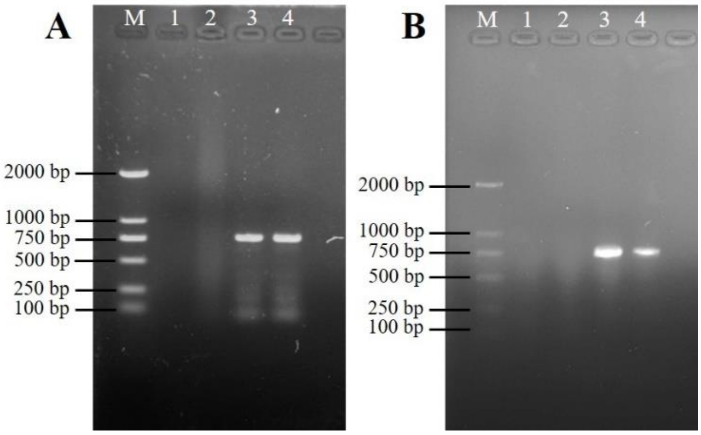

Figure S4. PCR analysis of the transformation of BjCHI1/ZmPR4 promoter-GUS in transgenic callus.

A: PCR check of BjCHI1 promoter-GUS in transgenic callus; B: PCR check of ZmPR4 promoter-GUS in transgenic callus; M: Marker 2000; 1, 2: non-transformed callus; 3, 4: representative transgenic callus.
