# Supplementary material for: Establishment of Highly Efficient Plant Regeneration, Callus Transformation and Analysis of Botrytis cinerea-Responsive PR Promoters in Lilium brownii var. viridulum
Source: Plants (Basel). 2023 May 16;12(10):1992. doi: 10.3390/plants12101992 (PMC10221712; doi:10.3390/plants12101992)
Supplement: Supplementary file 1 [file plants-12-01992-s001.zip › plants-2298569-supplementary tables.pdf]

Supplementary Tables:

Table S1 Effect of 6-BA and NAA combinations on the proliferation of embryogenic calli

MS/ (mg·L^{-1})		Callus number	Number of proliferated embryogenic callus	Frequency of proliferated embryogenic callus (%)	Multiplication index of embryogenic callus	Callus features	
6-BA	NAA					Texture	Color
0.1	0.1	50.00 ± 0.00 a	13.67 ± 2.40 d	27.33 ± 4.81 d	2.45 ± 0.23 c	Granular, friable	Pale yellow-white
0.5	0.1	50.00 ± 0.00 a	16.67 ± 2.33 cd	33.33 ± 4.67 cd	2.99 ± 0.10 c	Granular, compact	Pale yellow-white
0.1	0.5	50.33 ± 0.33 a	21.67 ± 0.88 bc	43.05 ± 1.73 bc	3.07 ± 0.01 c	Granular, friable	Pale yellow-white
0.5	0.5	50.33 ± 0.33 a	23.33 ± 1.76 b	46.33 ± 3.28 b	2.77 ± 0.14 c	Granular, compact	Light yellow
0.1	1.0	50.67 ± 0.67 a	18.33 ± 0.88 bcd	36.15 ± 1.29 bcd	3.02 ± 0.10 c	Granular, compact	Pale yellow-white
0.5	1.0	52.67 ± 2.67 a	29.67 ± 1.76 a	56.30 ± 1.19 a	3.96 ± 0.16 b	Granular, friable	Light yellow
*0.5	1.0	50.00 ± 0.00 a	32.33 ± 1.76 a	64.67 ± 3.53 a	5.19 ± 0.64 a	Granular, friable	Light green-purple

Table S2 Effects of different TDZ and NAA combinations on the shoot proliferation

MS/ (mg·L^{-1})		Callus number	Proliferated shoots number	Total shoot proliferation number	Proliferated shoots rate (%)	Shoot proliferation coefficient	Shoots features	
TDZ	NAA						Texture	Color
0.5	0.2	30.00	28.00±1.00 a	56.33±0.33 c	93.33±3.33 a	1.88±0.01 c	Partial short, Partial long	Green
0.5	0.5	30.00	27.00±2.52 a	65.00±6.43 bc	90.00±8.39 a	2.17±0.21 bc	Most short, less long	Tender green or green
1.0	0.2	30.00	28.33±1.67 a	57.33±8.17 c	94.44±5.56 a	1.91±0.27 c	Most short, less long	Tender green or green
1.0	0.5	30.00	29.00±1.00 a	91.33±4.10 a	96.67±3.33 a	3.28±0.25 a	Partial short, Partial long	Green
2.0	0.2	30.00	26.00±1.73 a	80.00±6.81 ab	86.67±5.77 a	2.67±0.23 ab	Partial short, Partial long	Green
2.0	0.5	30.00	28.33±0.33 a	76.67±5.78 ab	94.44±1.11 a	2.56±0.19 bc	Partial short, Partial long	Green

Table S3 Effects of different antibiotic concentration on the callus browning

Antibiotics	Concentration/ (mg·L^{-1})	Callus number	Callus browning number	Callus browning frequency (%)	Callus growth state
Cef	0	30	2.33 ± 0.33 a	8.00 ± 1.00 a	Grow well, rapid proliferation
	100	30	4.33 ± 0.67 ab	14.67 ± 2.33 ab	Less browning, normal proliferation
	200	30	4.33 ± 0.88 ab	14.33 ± 2.96 ab	Less browning, normal proliferation
	300	30	6.67 ± 0.33 bc	22.00 ± 1.00 bc	A little browning, decreased proliferation
	400	30	8.00 ± 0.58 cd	26.67 ± 2.03 cd	A little browning, decreased proliferation
Kan	0	30	1.33 ± 0.33 a	4.33 ± 1.33 a	Grow well, rapid proliferation
	50	30	1.33 ± 0.33 a	4.33 ± 1.33 a	Grow well, rapid proliferation
	75	30	1.67 ± 0.33 a	5.67 ± 1.33 a	Grow well, rapid proliferation
	100	30	2.33 ± 0.33 a	8.00 ± 1.00 a	Little browning, normal proliferation
	200	30	4.33 ± 0.33 ab	14.33 ± 1.33 ab	Less browning, decreased proliferation
	300	30	9.67 ± 0.88 de	32.67 ± 2.96 de	Partial browning, decreased proliferation
Hyg	0	30	2.00 ± 0.58 a	6.67 ± 2.03 a	Grow well, rapid proliferation
	25	30	14.00 ± 2.08 e	46.67 ± 6.94 e	Partial browning, decreased proliferation
	50	30	22.67 ± 2.40 f	75.56 ± 8.01 f	More browning, slight proliferation
	75	30	29.00 ± 0.58 g	96.67 ± 1.93 g	Almost browning, no proliferation
	100	30	29.67 ± 0.33 g	98.89 ± 1.11 g	All browning, no proliferation

Table S4 Summary of the induced expression of *GUS* gene

PR promoters	Treatments	Induced callus number	GUS-stained number	Induced expression efficiency (%)	Control callus number	GUS-stained number	Induced expression efficiency (%)
BjCHI1	1	14	10	71.43	16	1	6.25
	2	18	14	77.78	12	0	0
	3	14	7	50	10	0	0
ZmPR4	1	16	2	12.5	12	0	0
	2	12	0	0	11	0	0
	3	12	1	8.33	12	0	0
